# The changing malaria landscape in Aseer region, Kingdom of Saudi Arabia: 2000–2015

**DOI:** 10.1186/s12936-016-1581-2

**Published:** 2016-11-08

**Authors:** Ali Mohamed Alshahrani, Tarig M. Abdelgader, Ibrahim Saeed, AbdulRhman Al-Akhshami, Mohamed Al-Ghamdi, Mohammed H. Al-Zahrani, Ibrahim El Hassan, David Kyalo, Robert W. Snow

**Affiliations:** 1 Vector Control Administration, Aseer Health Affairs Directorate, Abha, Kingdom of Saudi Arabia; 2 Aseer General Directorate of Health Affairs, Abha, Kingdom of Saudi Arabia; 3 Public Health Directorate, Ministry of Health, Riyadh, Kingdom of Saudi Arabia; 4 Public Health and Tropical Medicine, University of Jazan, Jazan, Kingdom of Saudi Arabia; 5 Spatial Health Metrics Group, Kenya Medical Research Institute-Wellcome Trust Research Programme, Nairobi, Kenya; 6 Nuffield Department of Clinical Medicine, Centre for Tropical Medicine & Global Health, University of Oxford, Oxford, UK

## Abstract

**Background:**

In 2004, a revised action plan was developed, supported by the World Health Organization, to eliminate malaria from Saudi Arabia by preventing re-introduction of malaria into regions since declared malaria free, eliminating foci of transmission in the Mecca and Medina areas and a concerted effort of foci surveillance and control, to eliminate malaria from the regions of Jazan and Aseer. This paper provides the context, activities, progress, and possible contributions toward malaria elimination in the Aseer region since 2000, with a more detailed analysis of the spatial location of locally acquired case incidence since 2012.

**Methods:**

This is a descriptive study of all available Ministry of Health surveillance data and process reports since 2000, with higher spatial resolution analysis of data between 2012 and 2015.

**Results:**

In 2000, there were 511 cases of *Plasmodium falciparum* locally acquired infection. The following 4 years witnessed a dramatic decline in cases to only 18 locally acquired infections reported in 2005. A resurgence in local infections was reported in 2006 (93) and 2007 (165), thereafter (2008–2014) local cases continued to decline to fewer than 40 per year across the region. However, in 2015, a small rise was noted (51). All locally acquired infections were *P. falciparum*. There has been a constant flow of imported infections into Aseer since 2000, mostly among immigrant labour from Pakistan, India, Sudan, and Yemen. Imported infections have included both *Plasmodium vivax* and *P. falciparum*. The spatial extent of malaria appears to be changing, but there remain two intractable areas Sarat Abeda and Dhran Aljanub, where risks per reporting centre have changed little since 2001, remaining above 0.5 per 10,000 population. Only seven villages contributed 55% of all locally acquired infection since 2012.

**Discussion:**

Aseer has reached a state of very low incidence of locally acquired infections, despite a constant source of imported infections from outside the country. How many of the local infections are F2 generations from imported infections or how many are a result of residual active transmission between asymptomatic carriers of infections transmitted by pockets of existing *Anopheles arabiensis* populations remains unknown. A more detailed investigation of the spatial and temporal patterns of infected hosts, parasites and vectors would help define whether this region has managed to effectively prevent local transmission of new infections.

## Background

The latest global strategy for malaria provides a technical framework to accelerate progress toward eradication by 2030, adopted by the World Health Assembly in May 2015 [1]. The revised technical strategy aims to eliminate malaria from a further ten countries by 2020. Since 2007, two (United Arab Emirates and Morocco) of the five countries certified malaria free have been part of the World Health Organization’s Eastern Mediterranean Region (WHO-EMR); in addition, three of the 13 countries worldwide that have reported zero indigenous cases in 2014 were from EMR (Iraq, Oman and Syria). Two further WHO-EMR countries, Iran and Saudi Arabia have witnessed dramatic reductions in malaria case incidence since 2000, to the point where transmission is now constrained to foci within small geographic ranges.

For 20 years, following the launch of Saudi Arabia’s elimination programme, rapid progress was made in shrinking the geographic extent of malaria nationwide [2–4]. However, by the 1980s malaria remained an intractable problem in the southwestern regions of Jazan and Aseer. Emerging chloroquine resistance [5] and El Niño rains during the late 1990s led to severe epidemics in this region [2–4]. In 1998, the Government substantially increased the funding required to contain the epidemics in the southwestern regions of the Kingdom. In 2004, a revised action plan was developed, supported by WHO, to eliminate malaria from the Kingdom, the first statement of an elimination ambition since 1977. The focus of the revised plan was on preventing re-introduction of malaria into regions since declared malaria free, eliminating foci of transmission in the Mecca and Medina areas and a concerted effort, using foci surveillance and control, to eliminate malaria from the regions of Jazan and Aseer. Details of progress made in the Jazan region to 2014 have been presented elsewhere [6]. This paper describes activities, progress and possible contributions towards malaria elimination in the Aseer Region since 2000, with a more detailed analysis of the spatial location of locally acquired case incidence since 2012.

## Methods

### Location

**Fig. 1:**
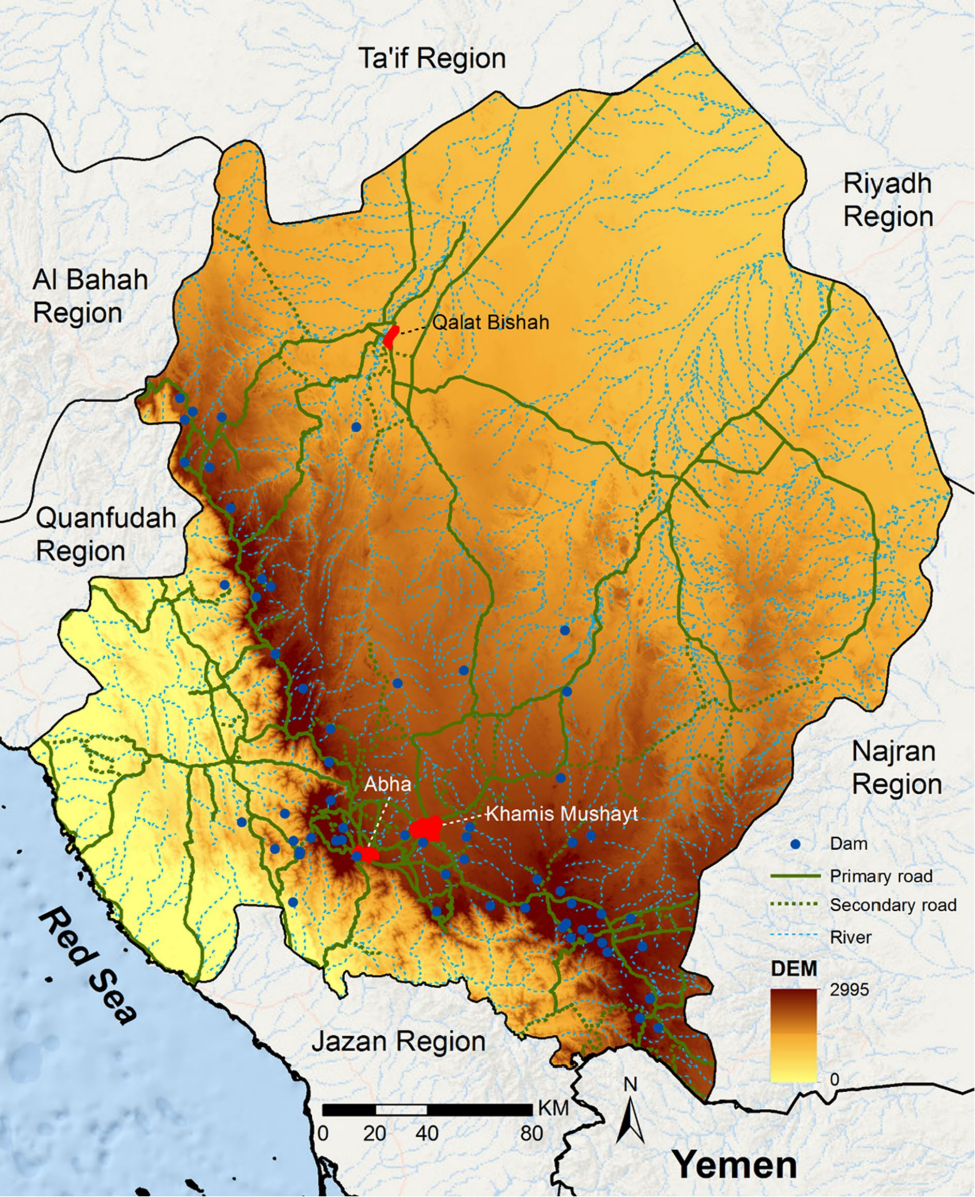
Topography of Aseer Region. Seasonal streams, dams and road network, major towns and digital elevation (masl) of Aseer Region

Aseer Region is located in the southwest of Saudi Arabia. It covers over 80,000 sq km of diverse topography, including the Tihama lowland plains that extend towards Yemen in the south and borders the Red Sea to the west; these plains rise to the mountainous areas of Sarawat that provide a network of seasonal streams, coursing through valleys that flood the lowland areas during the rains. East of the mountain spine descends to the arid Aseer Plateau (Fig. 1). The region has enjoyed rapid economic development since 2000, transforming some of the larger towns into modern cities, including Abha (the capital), Khamis Mushayt and Qalat Bishah (Fig. 1). According to the national census of 2010, 1.8 million people live in the province [7].

Rainfall is acutely seasonal with most precipitation occurring mid-May to mid-August, however, the amount of rainfall varies across the province depending on altitude, averaging between 300 and 500 mm each year. The river system feeds into wadis and dams across the region allowing for more perennial access to water for farming (Fig. 1). Terraced and valley agriculture has been the major occupation for many years representing one of the most fertile and agriculturally productive regions of the Kingdom, producing wheat, coffee, cotton, indigo, ginger, vegetables, and dates. Communities also raise cattle, sheep, goats, and camels.

### Malaria ecology

Aseer Region belongs to the Afro-tropical malaria ecological strata. Malaria transmission is maintained almost exclusively by *Anopheles arabiensis,* occasionally by *Anopheles sergentii* [8–13]. Other potential vectors have been identified in the province, *Anopheles culicifacies var. adenensis* and *Anopheles d’thali* [9–13]. However, their precise role in malaria transmission remains unknown.

**Fig. 2:**
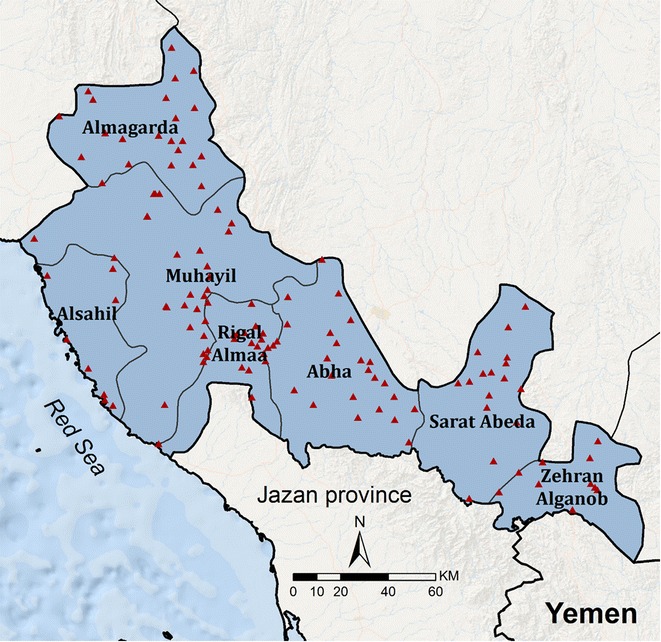
Malaria-reporting centres. Boundaries of malaria-reporting centres within the limits of malaria transmission in Aseer Region and the location of diagnostic PHCC facilities (*red triangles*)

In 1977, 209 people were surveyed for malaria infection across the Aseer Region [11]. The survey identified 42 (20%) *Plasmodium falciparum*, one *Plasmodium vivax* and two *Plasmodium malariae* infections in eight of the 14 villages investigated. During the early 1980s, malaria transmission was identified in highland areas of Zehran Aljanob, which prompted intensive anti-malarial treatment, larviciding and spraying activities to target adult *Anopheles gambiae* populations. In 1984, a pocket of indigenous vivax cases was identified in Tathleth, transmitted by *An. sergentii*, which was eliminated through vector control, large-scale mass-blood surveys and treatment. Since the 1990s, the semi-arid areas, located in the Aseer Plateau, the high altitude (>2000 m) areas and the larger towns of Abha and Khamis Mushayt have been regarded as malaria free. The spatial extent of malaria transmission since 2000 covers seven malaria-reporting centres (Fig. 2).

### Malaria control

Malaria control began in earnest in the Aseer Region during the early 1980s, following the establishment of a malaria centre at Abha in 1978 [14]. During this period the emphasis was on identifying malaria foci, parasitological investigation among school children and endemic villages in the Tihama lowland areas, bi-annual indoor residual house-spraying (IRS) using dichloro-diphenyl-trichloroethane (DDT) (until 1987), passive case detection and limited larviciding (temephos 50 EC, Abate) in selected areas of Tihama Qahatan. In 1984, larviciding expanded to cover all Tihama areas for 9 months in a year and adulticide activities to cover the remaining period of each year.

By 1986, the malaria programme was integrated into the regional Primary Health Care system, but technically supported by the Vector Borne Disease Administration (VBD). Activities continued to focus on passive case-detection, IRS with fenitrothion (1987–1991), pirimiphos- methyl (1992–1994), lambda-cyhlothrin (1995–2001) and deltamethrin (2002–2015), ultra-low volume (ULV) space spraying using a combination of Geothrin Plus (S-cyphenothrin 60% *plus* D-tetramethrin 40%), and cyfllulod PP (combination of cyfluthrin 1.5%, piperonlbutoxide 5%, pynamin forte 0.3%), larviciding using temephos 50 EC until 2004 thereafter using diflubenzuron 10% and/or 6%, with occasional seasonal use of poly (cxy-12 ethanediylalpha), and increasingly the provision of insecticide-treated nets (ITNs).

For many years the treatment policy was chloroquine (CQ), plus primaquine as a single dose to treat uncomplicated falciparum malaria and CQ followed by a 14 day regimen of primaquine for *P. vivax* and *Plasmodium ovale*. Second-line treatment for *P. falciparum* cases, in the event of CQ failure, was a single dose of sulfadoxine-pyrimethamine (SP). Cases of complicated malaria were treated with quinine. However, evidence of widespread CQ resistance was described during the late 1990s in neighbouring Jazan Region [15–17]. There was an increasing use of SP during periods of declining CQ efficacy, and artesunate (AS),plus, SP (AS/SP) and artemether-lumefantrine (AL) were also used during this period. An official change in treatment policy occurred in 2007 to support the use of AS/SP as the first-line treatment for uncomplicated malaria and AL as the second-line treatment [18]. For severe malaria, the recommended treatment options are presently either parenteral AS, artemether or quinine. New treatment policies were rolled out across the Aseer Region between 2007 and 2008 supported by booklets, posters, and distributed to health care providers in all health facilities, including public and private health facilities. Parasitological diagnosis of malaria has been the bedrock of clinical management since the 1990s; by 2004 rapid diagnostic tests (RDTs) were rolled out and became more widely used by the most peripheral of facilities.

By 1998, the malaria programme was fully funded by the Saudi Government through a special budget that allowed major scale-up of activities until the launch of the pre-elimination strategy in 2004. The role of the VBD administration is directly under the deputy Director General of Public Health, General Directorate of Health Affairs in Aseer Region. Under the new national pre-elimination strategy, roles include: setting standards, technical guidelines and quality assurance; training and capacity building; strengthening intra-sectoral collaboration and resource mobilization; leading activities to detect, prevent and control epidemics at the region level; guiding and implementing applied research activities; and, supervising, monitoring and evaluation of malaria control activities throughout the Aseer Region.

### Malaria surveillance

Malaria in the Kingdom was made a notifiable disease in 1959. With a view towards elimination, malaria case detection systems were increasingly strengthened from 1998. Since 2004, combinations of passive case detection (PCD) and active case detection (ACD) have been adopted through networks of primary health care centres (PHCCs) and notifications from the private sector. Cases are notified to malaria centre teams within 24 h via fax, WhatsApp or a 977 SMS text messaging service. Case investigations occur within three days of notification by VBD teams based in reporting centres, shown in Fig. 2. Teams investigate the origins of the infection through case and travel histories, screen community members within a 500 m radius of the index case, and undertake entomological investigations. Areas where locally acquired infections are registered become classified as foci for subsequent targeted intervention, larviciding and deltamethrin IRS (two rounds for the 6 month season in Tihama plains and three rounds for the 8 month season in the south and southeast of Aseer) and entomological surveillance for a further 12 months.

In theory, foci are classified as either residual active foci, where transmission has been defined within the last 2 years or new active foci, where transmission has started despite no previous reported transmission or 2 years of recession, and could be defined when only the first generation of transmission has taken place (i.e., only introduced cases are present) or secondary where introduced cases lead to second and subsequent indigenous cases.

For the purposes of presentation, data have been summarized in this paper by imported or locally acquired infection, month and by malaria reporting centre from January 2000 to December 2015. In addition, case data from the index case notification forms have been analysed in more detail for the 4 year period 2012 to 2015.

### Ethics statement

Data used in the present paper are derived from routine surveillance data of the Ministry of Health. Patient names have been anonymised. Data are not subject to ethical review as they form part of routine analysis of malaria elimination activities in the Aseer Region.

## Results

### Declining *Plasmodium falciparum* locally acquired case incidence 2000–2015

**Fig. 3:**
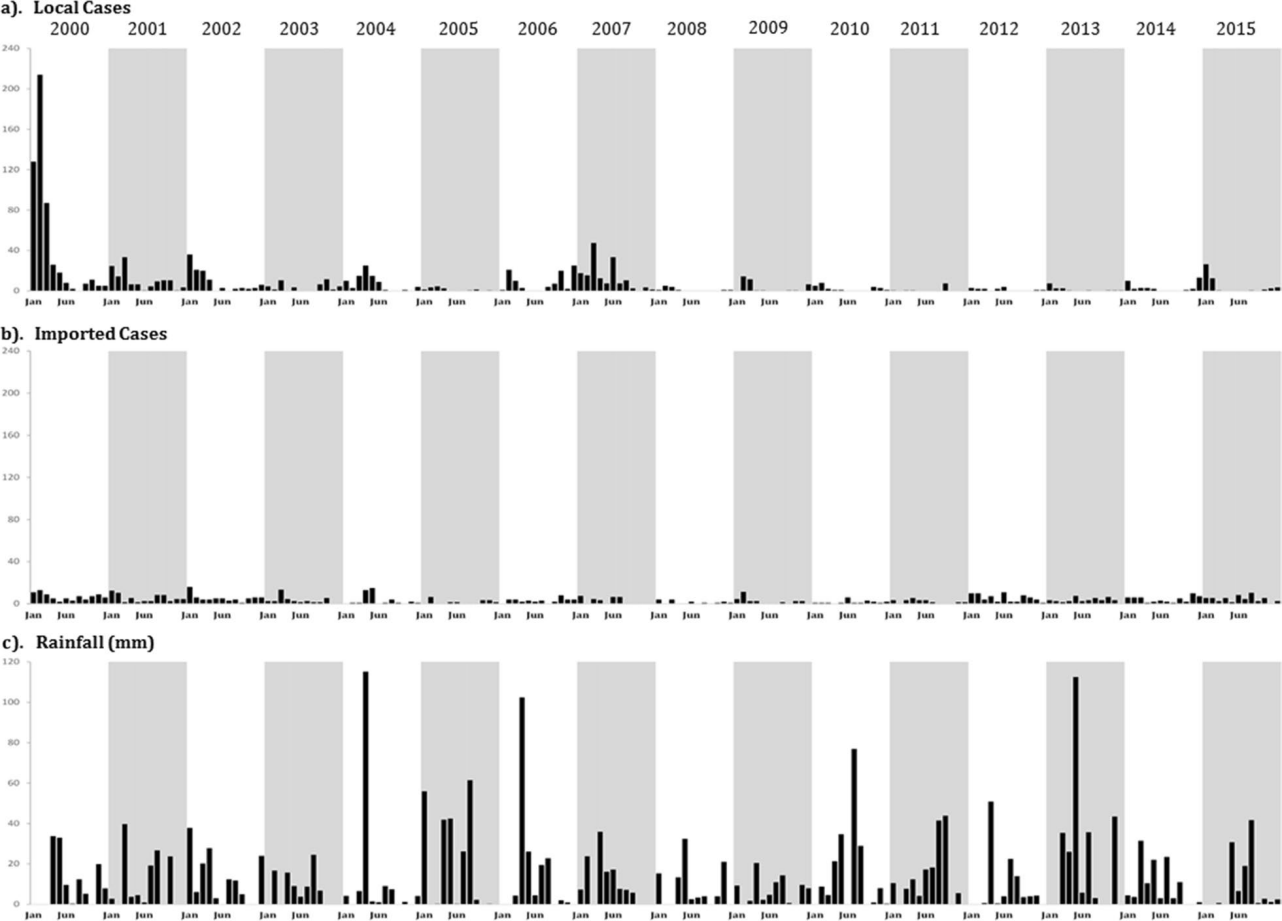
Monthly locally acquired malaria cases (**a**), imported cases (**b**) and rainfall (recorded in mm at Abha) (**c**) from January 2000 to December 2015

In 2000, there were 511 cases of *P. falciparum* locally acquired infection among residents of communities located in Aseer (Fig. 3). This exceptional peak occurred at a time when excessive rains led to an epidemic of Rift Valley Fever [19]. During the following 4 years cases dramatically declined to fewer than 140 each year, until 2005 when only 18 locally acquired infections were reported. Cases increased in 2006 (93) and 2007 (165) (Fig. 3). Between 2008 and 2014, fewer than 40 locally cases each year were detected from residents of Aseer. However, in 2015, 51 locally acquired cases of *P. falciparum* infection were detected from the region (Fig. 3). Since 2000, fewer than 90 cases per year of malaria described as imported have been detected in Aseer; the lowest years were 2008 (15) and 2010 (20) and the highest years were 2000 (81), 2001 (71) and 2012 (67) (Fig. 3).

**Fig. 4:**
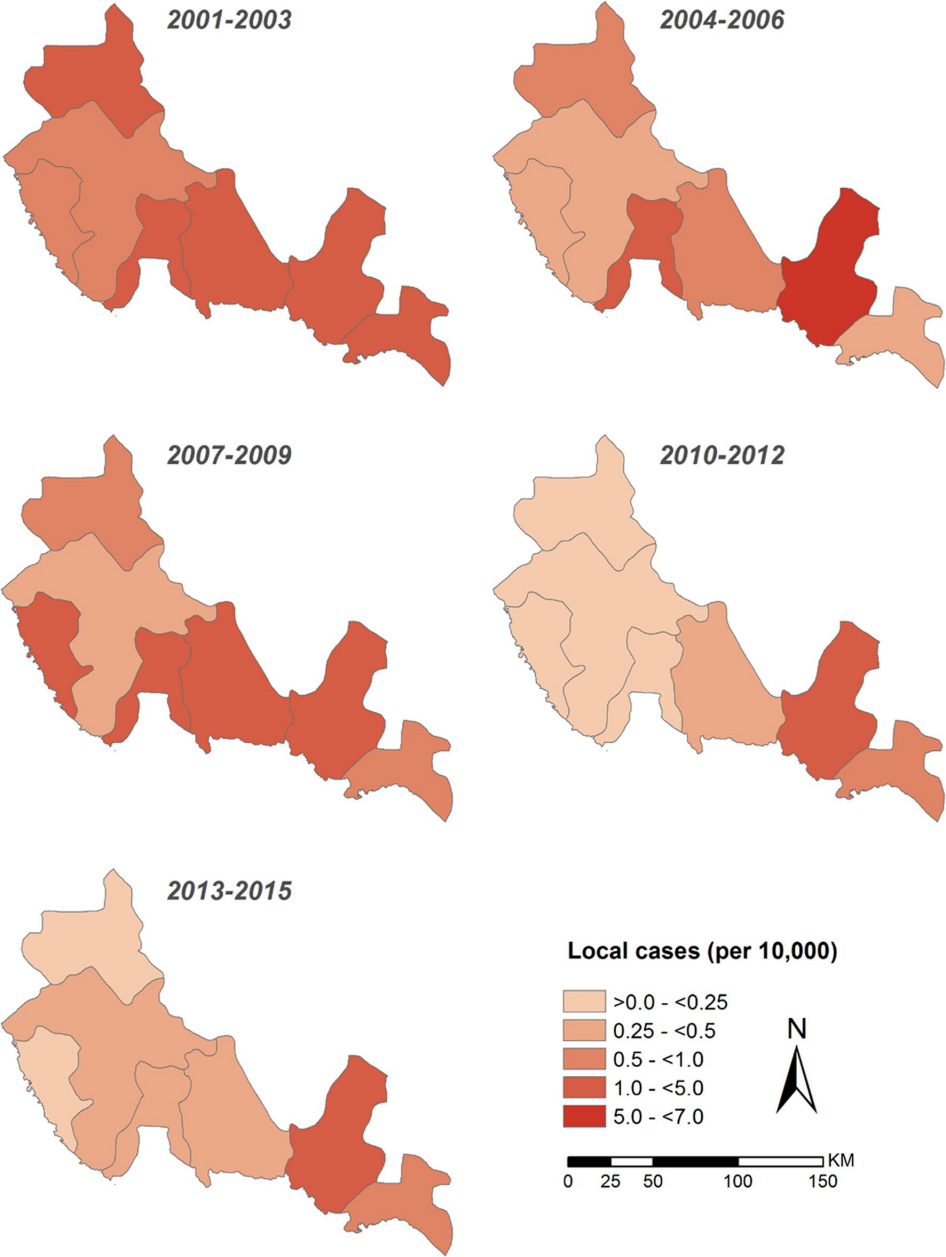
Average annual *Plasmodium falciparum* locally acquired case-incidence per 10,000 population

For each of the seven malaria-reporting centre catchment areas, populations at risk have been computed from village census data recorded during the 2004 and 2010 national censuses and projected backwards and forwards based upon intercensal annual growth rates. Figure 4 shows the average annual reported *P. falciparum* locally acquired case incidence for each centre for 3 year intervals, beginning in 2001 and excluding the anomalous epidemic year 2000. The average annualized reported incidence per malaria-reporting centre reduced in the five most northerly centres from 2009, to reporting rates of fewer than 0.5 per 10,000 people per year over the periods 2010–2015. Conversely, the two reporting regions of Sarat Abeda and Zehran Aljanob have changed little in their average reporting incidence since 2001, remaining above 0.5 per 10,000 population between 2010 and 2012 and 2013–2015 (Fig. 4).

### Spatial distribution of locally acquired cases 2012–2015

Figure 4 represents a crude annualized incidence per reporting centre. Before 2012, precise details of village residence were not geo-located using global positioning systems (GPS) or reliable documentation of exact location. This changed in 2012 to include the use of GPS and more detailed mapping during the case investigation procedures. Details from case-notification records have been reviewed for each reported malaria case since January 2012.

**Fig. 5:**
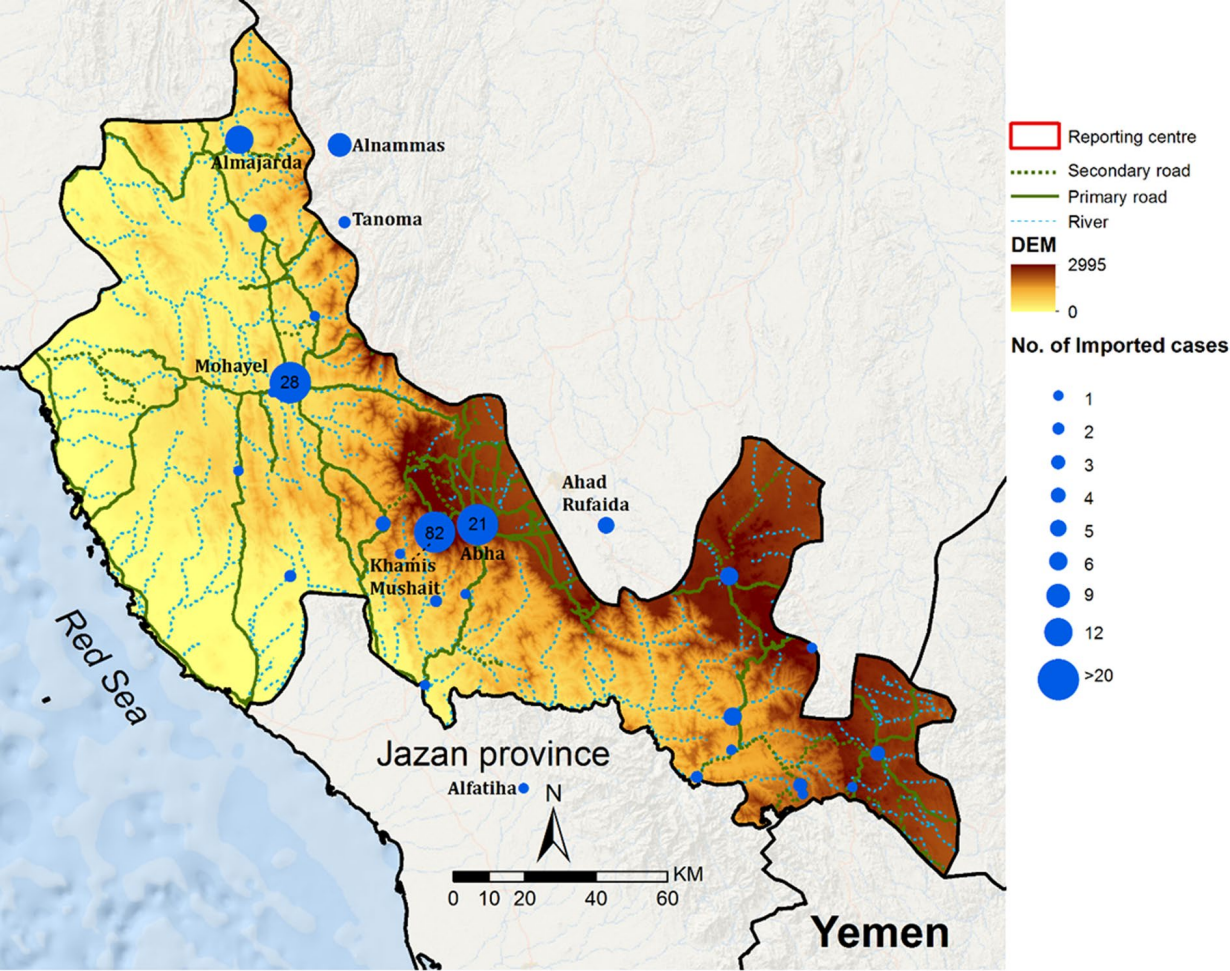
Village residences of infections classified as imported between January 2012 and December 2015. Size of *blue circle* represents numbers of cases over the entire period, as per legend

A total of 345 malaria cases were passively reported through the network of PHCCs across the seven malaria-reporting centres between January 2012 and December 2015. Case investigations established that 224 cases were infections imported from outside of the Kingdom (Table 1); these presented throughout the year without any obvious seasonal patterns (Fig. 3) and tended to be located in major towns (Fig. 5). Among the imported cases, 128 were pure *P. vivax* infections and two mixed infections with *P. falciparum*. Imported infections were predominantly among migrants of Pakistani or Indian origin (93), however it was notable that 20 imported *P. vivax* infections were detected among Sudanese immigrants, a country where vivax transmission is poorly described; less surprisingly, eight vivax infections were reported among Ethiopian immigrants (Table 1). Ninety-one *P. falciparum* imported infections were detected and two mixed infections with *P. vivax*. The majority of imported *P. falciparum* infections were among Yemenis (32), Saudis who had travelled to Yemen (3) and Ethiopians who had recently travelled from Yemen (14). Twenty-three *P. falciparum* infections were among Sudanese immigrants who had reported recent travel to Sudan (Table 1). Two cases of pure *P. malariae* infection were reported among two Sudanese patients with recent histories of home travel, noting that both are from Gezira (central Sudan). All imported *P. vivax* and *P. falciparum* infections were predominantly among adult males (Table 1).

**Table 1:** Characteristics of patients with *Plasmodium vivax* and *Plasmodium falciparum* infections acquired outside of the Kingdom between January 2012 and December 2015

	*P. vivax* (including 2 mixed infections) N = 130	*P. falciparum* (including two mixed infections) N = 93
*Nationality*		
Pakistani	67 (51.5%)	3 (3.2%)
Indian	26 (20.0%)	2 (2.2%)
Afghani	3 (2.3%)	0
Yemeni	4 (3.1%)	32 (34.4%)
Sudanese	21 (16.2%)	23 (24.7%)
Ethiopian	8 (6.6%)	26 (28.0%)
Kenyan	0	2 (2.2%)
Somali	0	1 (1.1%)
Saudi	1 (0.8%)^a^	4 (4.4%)^a^
*Males*	122 (93.9%)	86 (92.5%)
*Age (years)*		
<5	0	1 (1.1%)
5–14	3 (2.3%)	3 (3.3%)
15+	127 (97.7%)	89 (95.7%)

^a^Includes one patient who travelled to West Africa, with no other specific details of destinations, and acquired a mixed infection

**Fig. 6:**
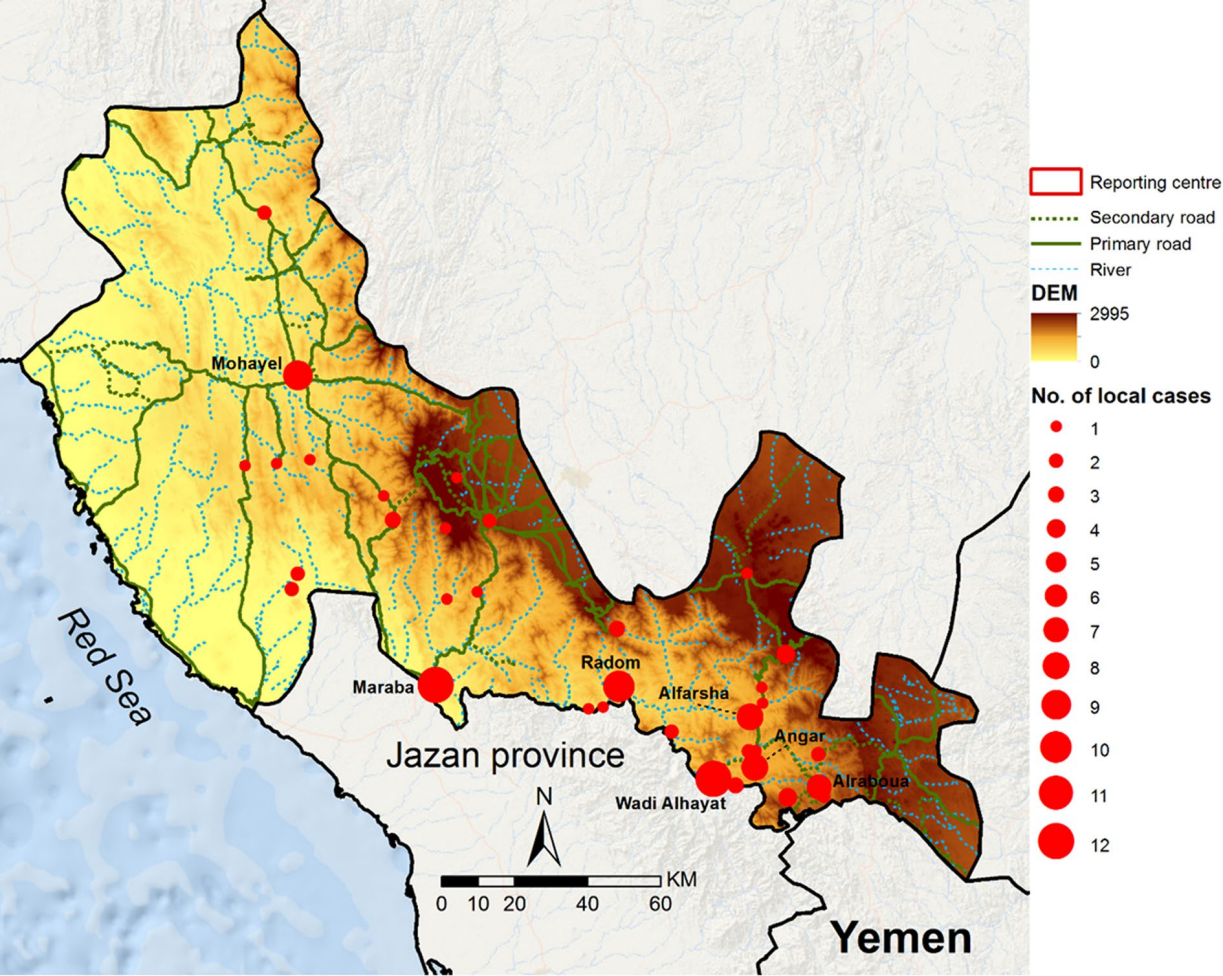
Village locations of locally acquired infections between January 2012 and December 2015. Size of *red circle* represents numbers of cases over the entire period, as per legend

One-hundred and twenty-one cases of *P. falciparum* infection were detected in Aseer and classified as being acquired in the Kingdom between 2012 and 2015. Among these locally acquired infections, 91 (75%) patients reported no recent travel outside their village of residence, of whom 79 were Saudi nationals, four were Yemeni nationals, three were Pakistanis, two were Sudanese, and three were Ethiopian. Of the non-Saudi nationals all had been resident in Aseer for over a year. Eleven patients reported recent travel to village locations within Aseer, 14 reported travel to neighbouring Jazan Region, one to Al Baha Region, three to Wadi Hammar in Yemen, and one who travelled to a destination that could not be defined. However, from travel histories each visit was for fewer than two days and were too recent for infections to have been acquired outside their residence in Aseer. Therefore, all 121 cases were defined as local infections likely to have been acquired in their residential homes in Aseer. Each residential village was validated using Google Earth and Open Street data (Fig. 6) for the entire period January 2012 to December 2015. Seven villages contributed 66 (54.6%) of all locally acquired cases between 2012 and 2015: Maraba (12), WadiAlhayat (12), Radom (10), Mohayel (9), Alfarsha (8), Angar (8), and Alraboua (7). In addition, two communities contributed four cases each, three communities contributed three cases each, eight communities contributed two cases each, 22 communities contributed one case across the surveillance period (Fig. 6). The majority of cases were located in communities close to the border with Jazan Region.

## Discussion

There have been significant declines in locally acquired malaria cases from 2000 in Aseer (Fig. 3). The case incidence is now exceptionally low, but not zero. It is notable that aberrations can occur with small rises in case incidence in some years, for example, 2007 and 2015. Reasons for these outbreaks are not clear, but cases are detected early and treated effectively. Large-scale onward transmission, to create seasonal epidemics typical of the 1990s, does not happen and there have been no deaths from malaria for over 25 years. While Aseer has not achieved elimination it has, nevertheless, achieved a public health success.

An important feature of this region over the last decade is that, against a background of sustained malaria control activities, there has been an equally significant increase in economic development. The landscape of paved roads, electrification of rural sectors, urbanization, and expansion of universal education and primary healthcare is completely different in the 2000s compared to the preceding decade. The impact of broader economic development cannot be uncoupled from malaria control success. Analysis of its direct contribution is beyond the scope of the present paper but warrants a more detailed investigation.

In concert with the notion of a changing receptive environment for low, stable *P. falciparum* transmission, a feature of Aseer a decade earlier, is that there continued to be a constant influx of imported malaria infections into the region (Fig. 3). These imported cases comprised both *P. vivax* and *P. falciparum* and originated from adults of non-Saudi nationality (Table 1) who presumably acquired their infections while in their native countries. The cases shown in Fig. 3 and Table 1 are those that have presented to PHCC diagnostic centres; because of their number, it is reasonable to assume that equivalent, if not more, infections are brought into the region by asymptomatic carriers who have an acquired clinical immunity. Despite this constant source of imported infection, the reported local residence of these cases (Fig. 5) appears not to correspond directly with the foci of locally acquired cases (Fig. 6), and equally there was no locally acquired *P. vivax* case in the region despite 57% of all imported cases harbouring this parasite. These observations are hard to explain based on the epidemiology of transmission. Vectors are not refractory to vivax in this area, and focal vivax epidemics have been reported previously. Addresses provided by imported cases may not be reliable, and it is possible that these cases migrate on further immediately after treatment. The lack of any obvious correspondence between imported infections and local infections may be a result of the overall change in social and land use ecology, resulting in a very different malariogenic potential or imprecision in the locality and duration of residence of imported cases.

The data presented here required a considerable degree of spatial confirmation. Cases reported within the catchment of one malaria-reporting centre had their household residence in another catchment area. This extended to cases detected in Aseer who were residents of villages in Jazan, and it was not possible to consider the reverse where cases reported in Jazan had residences in Aseer. Boundaries between malaria centres and between administrative regions (Jazan and Aseer) have also changed with time. A complete geo-coded census of all villages in either region is not available to the malaria programmes of these regions. Each residence requires confirmed coordinates each time a case presents to one of the PHCCs. These realities make the classification of area-specific incidence hard to define and interpret (Fig. 4). In addition, without an accurate, spatially configured denominator of all villages, meaningful analysis of hotspots cannot be undertaken [20–22], which would provide insights into putative risk factors [23–25]. Presently, and importantly when case incidence is so low, all that is possible is to map individual case residences within Aseer for those reported in Aseer. However, the residence of the host may not always be the same as the mosquito origin of the infection. Very detailed travel histories are required to establish precise locations where individuals may have slept over periods extending back at least ten days before they present with clinical symptoms. There may be social reasons why respondents might be reluctant to provide accurate information, including recreational travel. Even with reliable travel histories of those who have been locally mobile there is no guarantee that an infection can be linked to the travel location or the usual residence, even with current genotype barcodes of the parasite [26, 27]. Where genotyping parasites will become valuable is in the mapping of onward transmission. Being able to classify F1 and subsequent generations of new infections in an area would provide useful information for a programme to understand how foci evolve [28].

The malaria programmes in Aseer and Jazan have slightly different procedures for detecting cases; in Jazan, for example, annual mass blood surveys are undertaken in areas of previous foci. In Aseer, reactive ACD is mounted during the year an imported or locally acquired case is detected, however, during the following year communities in these localities are not screened again. The degree to which locations where cases are identified are temporally interrogated determines the reliable classification of active, residual non-active and cleared foci [28]. These definitions were proposed as metrics for targeted elimination in Aseer and Jazan during the Kingdom’s 2004 plan of action [29]. These definitions, based on case data, mass blood surveys and entomological investigations are not strictly adhered to and are not mapped in a way that allows time and space to be included in the 2 year cut-off periods between foci classifications. Nevertheless, with the available resources, and in the absence of a universal geographic information system (GIS), the notion of foci are used as part of timely notification of malaria cases from public and private sector health facilities, leading to every locality being investigated for the characteristics of transmission to mount intensified control. This pragmatic approach continues to serve Aseer well, and case incidence remains low. To reach a position where complete confirmation of the absence of transmission, for a certification of elimination, would however require a substantial increase in use of GIS, increased 2 year longitudinal, active surveillance of sites where cases have been detected, and a more detailed, coordinated interrogation of travel and residence histories within Aseer and with neighbouring Jazan.

## Conclusion

Aseer has reached a state of exceptionally low incidence of locally acquired infections, despite a constant source of imported infections from outside the country. How many of the local infections are F2 generations from imported infections or how many are a result of residual active transmission between asymptomatic carriers of infections transmitted by pockets of existing *An. arabiensis* populations remains unknown. Over the past decade it is possible that the region’s economic development has changed the receptive nature of malaria in this area; imported infections are less likely to find the possibility for onward transmission and the rapidity in detecting and treating cases has reduced the infective parasite biomass. A more detailed investigation of the spatial and temporal patterns of infected hosts, parasites and vectors would help define whether this region has managed to effectively prevent local transmission of new infections.

## Abbreviations

ACD: active case detection
AS/SP: artesunate plus sulfadoxine-pyrimethamine
AL: artemether-lumefantrine
CQ: chloroquine
DDT: dichloro-diphenyl-trichloroethane
F2: second generation infections
GIS: geographic information systems
GPS: global positioning system
IRS: indoor residual house-spraying
ITN: insecticide-treated nets
PCD: passive case detection
PHCC: primary health care centre
RDT: rapid diagnostic tests
ULV: ultra-low volume
VBD: vector borne disease administration
WHO-EMR: World Health Organization’s Eastern Mediterranean Region
